# Supplementary material for: The Effects of a Comprehensive, Integrated Obesity Prevention Intervention Approach (SuperFIT) on Children’s Physical Activity, Sedentary Behavior, and BMI Z-Score
Source: Int J Environ Res Public Health. 2019 Dec 10;16(24):5016. doi: 10.3390/ijerph16245016 (PMC6950277; doi:10.3390/ijerph16245016)
Supplement: Supplementary file 1 [file ijerph-16-05016-s001.pdf]

International Journal of
Environmental Research and Public Health

MDPI

Supplementary Material

Table S1. Sensitivity analysis of the effects of SuperFIT on PA using only cases with data on all measurements.

		Full intervention (N=17)*	Partial intervention (N=21)*	Control (N=43)	Full vs. control			Partial vs. control		
		Mean ±SD**	Mean ±SD**	Mean ±SD**	B[a] (95%CI)	P	ES	B[a] (95%CI)	P	ES
SB (%)	T0	81.39 ±5.42	81.15 ±3.74	81.09 ±5.98						
	T1	79.00 ±2.09	80.73 ±3.45	80.25 ±5.23	-1.54 (-3.93; 0.85)	0.21	-0.31	0.43 (-1.79; 2.65)	0.70	0.10
	T2	77.96 ±5.08[$]	77.64 ±4.27[$]	77.27 ±5.46[$]	0.40 (-2.08; 2.87)	0.75	0.08	0.33 (-1.97; 2.63)	0.76	0.07
LPA (%)	T0	9.65 ±2.06	10.03 ±1.32	9.31 ±2.43						
	T1	10.00 ±2.09	9.50 ±1.24	9.60 ±2.01	0.07 (-1.00; 1.14)	0.90	0.03	-0.82 (-1.81; 0.18)	0.11	-0.40
	T2	10.49 ±1.71	11.03 ±1.67[$]	10.62 ±1.70[$]	-0.46 (-1.58; 0.65)	0.41	-0.23	-0.31 (-1.34; 0.73)	0.56	-0.15
MVPA (%)	T0	8.97 ±3.51	8.82 ±2.66	9.59 ±3.96						
	T1	10.99 ±4.42[$]	9.77 ±2.43[$]	10.15 ±2.01	1.08 (-0.52; 2.69)	0,18	0.33	0.10 (-1.38; 1.58)	0.90	0.03
	T2	11.55 ±3.68[$]	11.31 ±3.17[$]	12.11 ±4.20[$]	-0.24 (-1.91; 1.43)	0.78	-0.07	-0.22 (-1.76; 1.32)	0.78	-0.07
CPM	T0	1111.35 ±284.12	1162.07 ±185.85	1169.62 ±329.18						
	T1	1206.63 ±321.45	1152.29 ±167.56	1190.83 ±279.39	74.07 (-52.83; 200.98)	0.25	0.28	-30.98 (-148.91; 86.94)	0.60	-0.12
	T2	1330.08 ±250.98[$]	1367.82 ±199.44[$]	1404.42 ±340.71[$]	-16.07 (-170.72; 138.58)	0.85	-0.17	-29.05 (-172.76; 114.66)	0.69	-0.11

*Full intervention= exposed to both preschool-based and family-based component, partial intervention= exposed to only preschool-based component **Observed scores [a]Linear mixed model analysis corrected for baseline, child age, child gender and weather (temperature, precipitation and sunshine) [$]Significantly different from baseline score, analysed with paired *t*-tests. CI= confidence interval, CPM= counts per minute, ES= effect size, LPA= light physical activity, MVPA= moderate-to-vigorous physical activity, SB= sedentary behaviour, SD= standard deviation.

Table S2. Sensitivity analysis of the effects of SuperFIT on BMI z-score including only cases with data on all measurements.

		Full intervention* (N=27)	Partial intervention* (N=26)	Control (N=58)	Full vs. control			Partial vs. control		
		Mean±SD**	Mean±SD**	Mean±SD**	B[a] (95%CI)[a]	P	ES	B[a] (95%CI)[a]	P	ES
BMI z-score	T0	0.34±1.06	0.19±0.86	0.19±1.03						
	T1	0.26±1.00	0.22±0.76	0.23±1.03	-0.11 (-0.37; 0.14)	0.37	-0.12	-0.00 (-0.26; 0.25)	0.99	0.00
	T2	0.26±0.90	-0.04±0.73[$]	0.17±0.92	-0.06 (-0.35; 0.22)	0.67	-0.06	-0.21 (-0.50; 0.08)	0.15	-0.22

*Full intervention= exposed to both preschool-based and family-based component, partial intervention= exposed to only preschool-based component **Observed scores [a]Linear mixed model analysis corrected for baseline, parental BMI, parental education and parental country of birth [$]Significantly different from baseline score, analysed with paired *t*-tests. BMI= body mass index, CI= confidence interval, ES= effect size, SD= standard deviation.
